# Neuregulin 1/ErbB4 signaling contributes to the anti-epileptic effects of the ketogenic diet

**DOI:** 10.1186/s13578-021-00536-1

**Published:** 2021-02-03

**Authors:** Jin Wang, Jie Huang, Yuan-Quan Li, Shan Yao, Cui-Hong Wu, Ying Wang, Feng Gao, Min-Dong Xu, Guo-Bin Huang, Chang-Qin Zhao, Jia-Hui Wu, Yun-Long Zhang, Renjie Jiao, Zi-Hao Deng, Wei Jie, Hui-Bin Li, Aiguo Xuan, Xiang-Dong Sun

**Affiliations:** 1 School of Basic Medical Sciences, Institute of Neuroscience and Department of Neurology of the Second Affiliated Hospital of Guangzhou Medical University, Key Laboratory of Neurogenetics and Channelopathies of Guangdong Province and the Ministry of Education of China, Guangzhou, 510260 China; 2 Department of Neurology of the Sixth Affiliated Hospital, Guangzhou Medical University, Guangzhou, 511518 China; 3 Institute of Life Science, Nanchang University, Nanchang, 330031 China; 4 Department of Physiology, School of Medicine, Jinan University, Guangzhou, 510632 China; 5 Sino-French Hoffmann Institute, School of Basic Medical Sciences, Guangzhou Medical University, Guangzhou, 511436 China; 6 Guangdong Province Key Laboratory of Psychiatric Disorders, Southern Medical University, Guangzhou, 510515 China; 7 Department of Pathology, Guangdong Women and Children Hospital, Guangzhou, 511400 China

**Keywords:** Neuregulin 1, ErbB4, Ketogenic diet, GABAergic activity, Epilepsy

## Abstract

**Background:**

The ketogenic diet (KD) has been recognized as a potentially effective therapy to treat neuropsychiatric diseases, including epilepsy. Previous studies have indicated that KD treatment elevates γ-Amino butyric acid (GABA) levels in both human and murine brains, which presumably contributes to the KD’s anti-seizure effects. However, this has not been systematically investigated at the synaptic level, and the underlying molecular mechanisms remain to be elucidated.

**Methods:**

Kainic acid (KA)-induced acute and chronic seizure models were utilized to examine the effects of KD treatment on seizure threshold and epileptogenesis. Synaptic activities in the hippocampus were recorded with the technique of electrophysiology. The effects of the KD on Neuregulin 1 (Nrg1) expression were assessed via RNA sequencing, real-time PCR and Western blotting. The obligatory role of Nrg1 in KD’s effects on seizures was evaluated through disruption of Nrg1 signaling in mice by genetically deleting its receptor-ErbB4.

**Results:**

We found that KD treatment suppressed seizures in both acute and chronic seizure models and enhanced presynaptic GABA release probability in the hippocampus. By screening molecular targets linked to GABAergic activity with transcriptome analysis, we identified that KD treatment dramatically increased the *Nrg1* gene expression in the hippocampus. Disruption of Nrg1 signaling by genetically deleting its receptor-ErbB4 abolished KD’s effects on GABAergic activity and seizures.

**Conclusion:**

Our findings suggest a critical role of Nrg1/ErbB4 signaling in mediating KD’s effects on GABAergic activity and seizures, shedding light on developing new therapeutic interventions to seizure control.

## Background

Epilepsy is a severe neurological disorder, characterized by recurrent behavioral seizures and abnormal synchronized neuronal firing. Despite the availability of numerous drugs in the clinic, approximately one-third of the patients develop resistance to these treatment regimens [1]. Identifying novel molecular targets for epilepsy will provide a framework to develop highly potent therapeutic strategies.

The ketogenic diet (KD) is rich in fat with adequate protein and low carbohydrates content and is known to induce a sustained ketotic state by producing heightened ketone bodies. Being a part of bioenergetics medicine therapies, the KD is frequently utilized either alone or in combination with other anti-seizure drugs to treat refractory epilepsy [2]. However, its application has been restricted due to, at least in part, dietary compliance and adverse side effects [3]. Understanding the critical cellular and molecular signaling mechanisms underlying the therapeutic effects of the KD will not only provide insights into mechanisms of epilepsy but also facilitate to develop effective pharmacological strategies. Previous studies have identified roles of the KD in the regulation of mitochondrial respiration, oxidative stress, inflammation, protein post-translational modifications, gut microbiota-brain communication, ion channel properties [4]. However, the exact molecular mechanisms through which the KD induces beneficial effects on controlling seizures remain obscure.

γ-Amino butyric acid (GABA) is the primary inhibitory neurotransmitter in the central nervous system. GABAergic transmission functions to integrate excitatory inputs, synchronize neuronal activities, control synaptic plasticity and prevent the generation and spread of paroxysmal activities [5]. A vast amount of evidence has demonstrated that deficiency in GABAergic functions could lead to epilepsy. Blockade of GABAergic transmission by antagonists of GABA receptors, including pentylenetetrazole (PTZ) and bicuculline, causes acute epileptic discharges, which has been widely used as experimental epilepsy models [6]. Likewise, various anti-epileptic drugs like benzodiazepines, barbiturates and tiagabine, are known to alleviate seizures by enhancing GABAergic inhibition [7]. Remarkably, numerous studies have reported that KD treatment elevated GABA levels but not excitatory acid-glutamate in both human and murine brains [8–10]. These observations suggest a potential role of the KD in the regulation of GABAergic activity in the brain. However, this has not been systematically investigated at the synaptic level, and the exact underlying regulatory mechanisms remain to be elucidated.

In the present study, we address these questions by using a combination of genetic, electrophysiological and biochemical tools. We find that KD treatment suppresses seizures in both acute and chronic seizure animal models and elevates the GABAergic but not glutamatergic activity in the hippocampus. By screening the molecular targets that potentially linked to GABAergic transmission through transcriptome analysis, we identify that the Nrg1 expression was dramatically increased by KD treatment. Disruption of Nrg1 signaling by genetically deleting its receptor-ErbB4 diminishes the effects of the KD on GABAergic synaptic transmission and seizures. Altogether, these findings suggest a critical role of Nrg1/ErbB4 signaling in mediating the effects of KD on GABAergic activity and seizure resistance, which sheds light on developing new therapeutic interventions to seizure control.

## Results

### The ketogenic diet exhibits protective effects in both acute and chronic seizure models

**Fig. 1:**
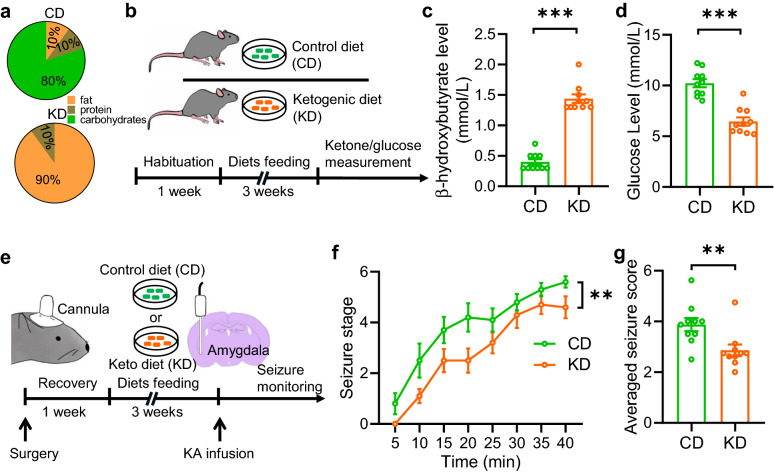
Ketogenic diet (KD) treatment elevates threshold to seizure in KA-induced seizure model. **a** Diagrams of diet contents. **b** Experimental design. After 1 week of habituation, male adult mice were divided into two groups, fed with control diet (CD) or KD for 3 weeks. Ketone and glucose measurements were then performed. **c** Increased circulating levels of β-hydroxybutyrate after 3 weeks of KD treatment. n = 10 mice per group. Student’s t test, t_(18)_ = 12.49, P < 0.0001. **d** Decreased circulating levels of glucose after 3 weeks of KD treatment. n = 10 mice per group. Student’s t test, t_(18)_ = 6.604, P < 0.0001. **e** Experimental design of seizure induction with kainic acid (KA). One week after cannula implantation into the amygdala, mice were fed with CD or KD. At the end of 3-weeks, mice were infused with kainic acid (KA) through cannula and seizure behaviors were monitored. **f** KD treatment decelerated the development of seizures. Mice in two groups were subject to KA infusion and scored for seizure stage every 5 min. n = 10 mice for each group. Repeated two-way ANOVA, F_(1,126)_ = 8.522, P = 0.0092. **g** The averaged seizure score was decreased in mice fed with KD. n = 10 mice for each group. Student’s t test, t_(18)_ = 2.919, p = 0.0092

To examine the effects of the ketogenic diet (KD) on seizure, we first fed adult male mice with the KD or composition-matched control diet (CD) for 3 weeks (Fig. 1a and b). The serum levels of β-hydroxybutyrate, a primary form of ketones, were markedly elevated in KD-fed mice compared with that in CD-fed mice (Fig. 1c). In contrast, we observed reduced glucose levels in mice fed with the KD (Fig. 1d). These results indicate that KD treatment successfully induces a state of increased ketone bodies in mice. In the acute seizure model, kainic acid (KA) was micro-infused into the amygdala to induce seizures (Fig. 1e). We found that the infusion of KA caused prolonged convulsive motor seizures in CD-fed mice. However, the seizures were dramatically decreased in KD-fed mice (Fig. 1f). Accordingly, the averaged seizure score was lower in KD-fed mice than in CD-fed mice (Fig. 1g). These data indicate that KD treatment results in a raised threshold to seizure.

**Fig. 2:**
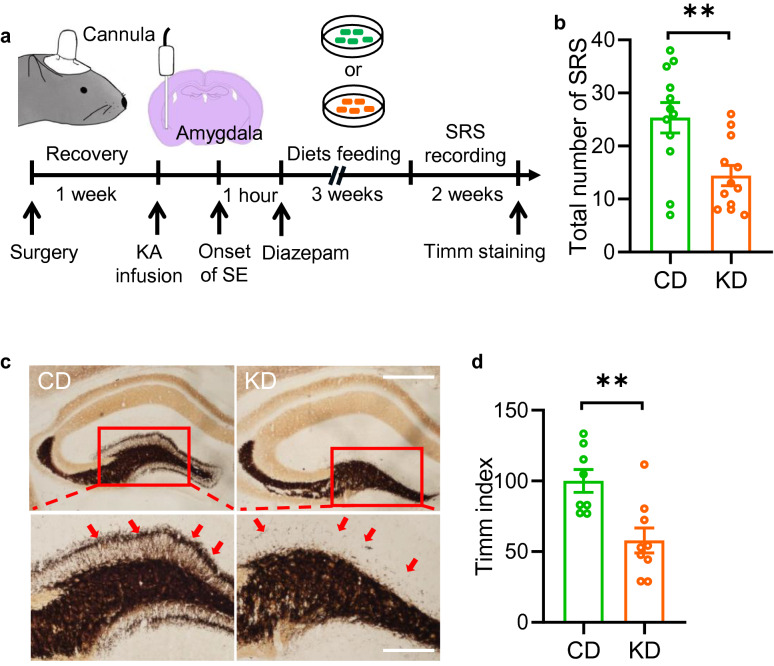
KD treatment suppresses epileptogenesis in KA-induced chronic seizure model. **a** Experimental design for spontaneous recurrent seizure (SRS) recording. SE was induced by the infusion of KA in the amygdala. One hour later, Seizure activity was terminated by injection of diazepam (i.p). Mice were then fed with CD or KD for 3 weeks, followed by 2 weeks of SRS recording. **b** reduced SRS number in mice fed with KD. n = 12 mice per group. Student’s t test, t_(22)_ = 3.186, P = 0.0043. **c** Representative Timm staining images of brain sections from mice fed with CD or KD Magnified images of areas in dotted squares were shown on the bottom. Arrows, Timm granules in the supragranular region. Scale bar, 500 µm (top) and 200 µm (bottom). **d** Statistical analysis of the Timm index. n = 8 slices from 3 CD-fed mice; n = 9 slices from 3 KD-fed mice. Student’s t test, t_(15)_ = 3.473, P = 0.0034

It has been widely reported that an episode of status epilepticus (SE) promotes the development of temporal lobe epilepsy in humans and mammalian animals [11]. To test whether KD feeding also prevents SE-induced spontaneous recurrent seizure (SRS), a typical process of epileptogenesis, we treated C57 mice with CD or KD after SE termination. Three weeks later, we analyzed the number of SRS in the following 2 weeks (Fig. 2a). We found that KD-fed mice exhibited a reduced number of SRS compared with that in CD-fed group, indicating a suppressive effect of the KD on epileptogenesis (Fig. 2b). Furthermore, we also examined the extent of the mossy fiber sprouting, a chronic pathological feature of limbic epilepsy, by using Timm staining. We found that the Timm index of mice fed with the KD was significantly lower than that in CD-fed mice (Fig. 2c and d), indicating a protective effect of KD treatment. Altogether, these observations suggest that the KD possesses both anti-convulsant and anti-epileptogenic effects.

### Ketogenic diet treatment increases inhibitory synaptic activity in the hippocampus

**Fig. 3:**
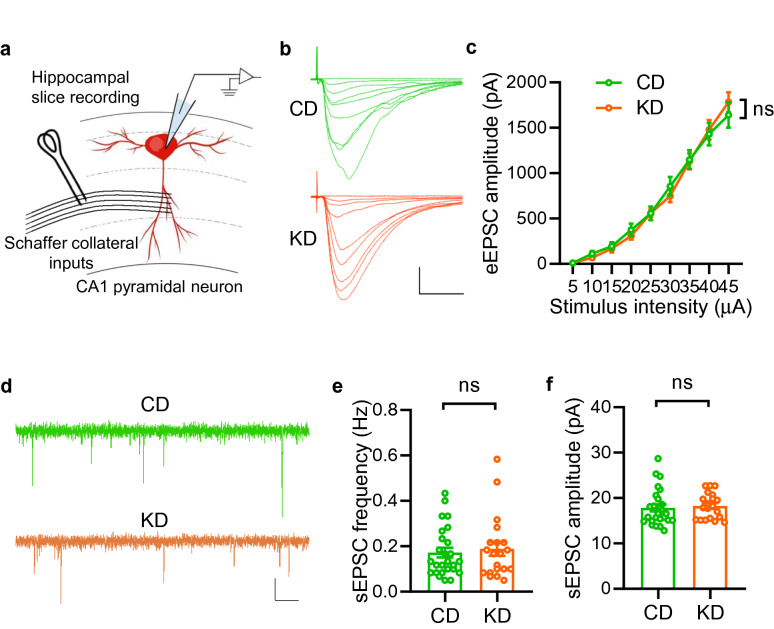
KD treatment exhibits little effect on excitatory synaptic transmission in the hippocampus. **a** Diagram of hippocampal slice recording. Pyramidal neurons in the CA1 region were clamped in whole-cell configuration. Evoked excitatory postsynaptic currents (eEPSCs) were recorded under the stimulation of Schaffer collateral inputs by using a concentric bipolar electrode. **b** Representative eEPSC traces. Scale bars, 20 ms and 500 pA. **c** Comparable amplitudes of eEPSCs in the CA1 region of hippocampus between CD and KD group. n = 28 neurons from 4 mice fed CD; n = 24 neurons from 4 mice fed KD. Two-way ANOVA, F_(1,450)_ = 0.05, P = 0.8231. **d** Representative sEPSC traces. Scale bars, 2 s and 10 pA. **e**, **f** Unaltered sEPSC frequency and amplitude between two groups. n = 26 neurons from 4 mice fed with CD; n = 20 neurons from 4 KD-fed mice. Student’s t test, for frequency, t_(44)_ = 0.4743, P = 0.6376;, for amplitude, t_(44)_ = 0.3979, P = 0.6926

Altered synaptic transmission, which accounts for unbalance of inhibition/excitation, is generally considered as a critical pathological mechanism of seizure occurrence [12]. To investigate the cellular mechanisms by which the KD affects seizure, we first recorded evoked excitatory postsynaptic currents (eEPSCs) from pyramidal neurons in the CA1 region of hippocampus, a critical brain area for epileptogenesis, by stimulating Schaffer Collaterals (SC) pathway (Fig. 3a). The eEPSC amplitudes in mice fed with the KD were comparable to those in CD-fed mice (Fig. 3b and c). Besides, we also found no differences in both frequency and amplitude of sEPSCs (Fig. 3d to f). These observations indicate that KD feeding plays a limited role in regulating excitatory synaptic transmission in the hippocampus.

**Fig. 4:**
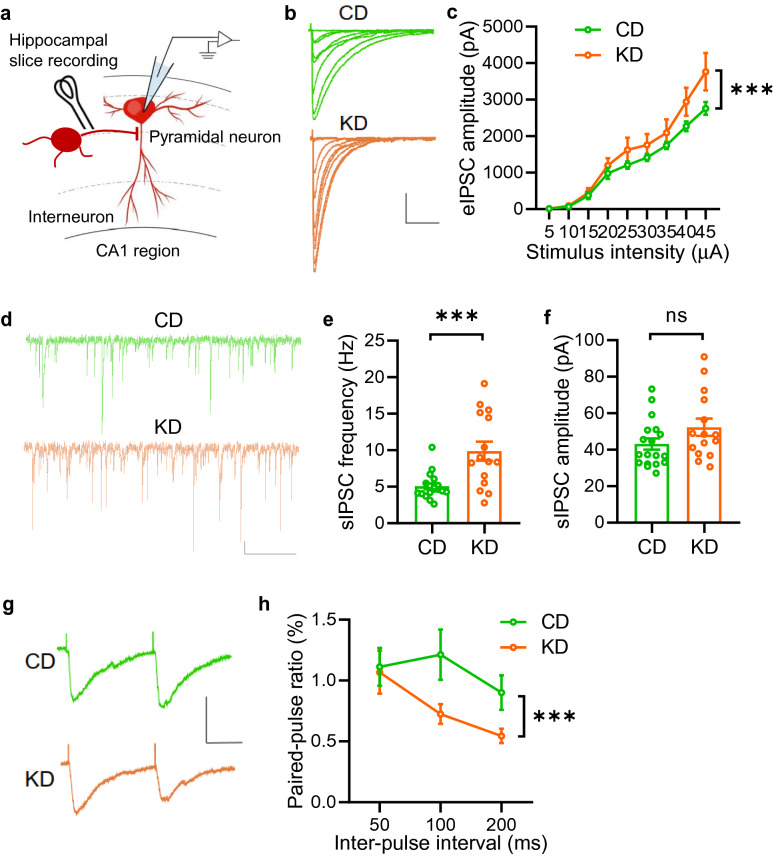
KD treatment increases inhibitory synaptic activity in the hippocampus. **a** Diagram of hippocampal slice recording. Pyramidal neurons in the CA1 region were clamped in whole-cell configuration. Evoked postsynaptic currents were recorded under stimulation by using a concentric bipolar electrode. **b** Representative eIPSC traces. Scale bars, 100 ms and 1000 pA. **c** Increased amplitude of evoked inhibitory postsynaptic currents (eIPSCs) in the CA1 region of hippocampus in KD-fed mice. n = 17 neurons from 4 CD-fed mice; n = 15 neurons from 4 KD-fed mice. Two-way ANOVA, main effect, F_(1,267)_ = 11.9, P = 0.0007. **d** Representative spontaneous inhibitory postsynaptic current (sIPSC) traces. Scale bars, 2 s and 20 pA. **e**, **f** Increased sIPSC frequency but not amplitude in KD-fed mice. n = 18 neurons from 4 CD-fed mice; n = 15 neurons from 4 KD-fed mice. Student’s t test, for frequency, t_(31)_ = 3.782, P = 0.0007; for amplitude, t_(31)_ = 1.678, P = 0.1034. **g** Representative sweeps with inter-stimulus interval of pair-pulse stimulations at 100 ms. Scale bars, 50 ms and 100 pA. **h** Decreased PPRs in KD-fed mice. n = 17 neurons from 4 CD-fed mice; n = 15 neurons from 4 KD-fed mice. Two-way ANOVA, F_(1,90)_ = 5.871, P = 0.0174

In contrast, we observed dramatically increased amplitude of evoked inhibitory synaptic currents (eIPSCs) in KD-fed mice compared with those in CD-fed mice (Fig. 4a to c). Moreover, the frequency but not the amplitude of sIPSCs was enhanced in KD-fed mice (Fig. 4d to f). To further investigate the possible involvement of presynaptic GABA release probability, we recorded paired-pulse ratios (PPR) in the CA1 region. We found decreased levels of PPR in KD-fed mice compared with those in CD-fed mice (Fig. 4g and h), indicating enhanced presynaptic GABA release probability. In conclusion, these results suggest that KD treatment enhances inhibition/excitation ratio in the hippocampus, mainly via increasing inhibitory synaptic transmission, which may underly the protective effects of the KD on seizures.

### KD treatment elevates Nrg1 expression in the hippocampus

**Fig. 5:**
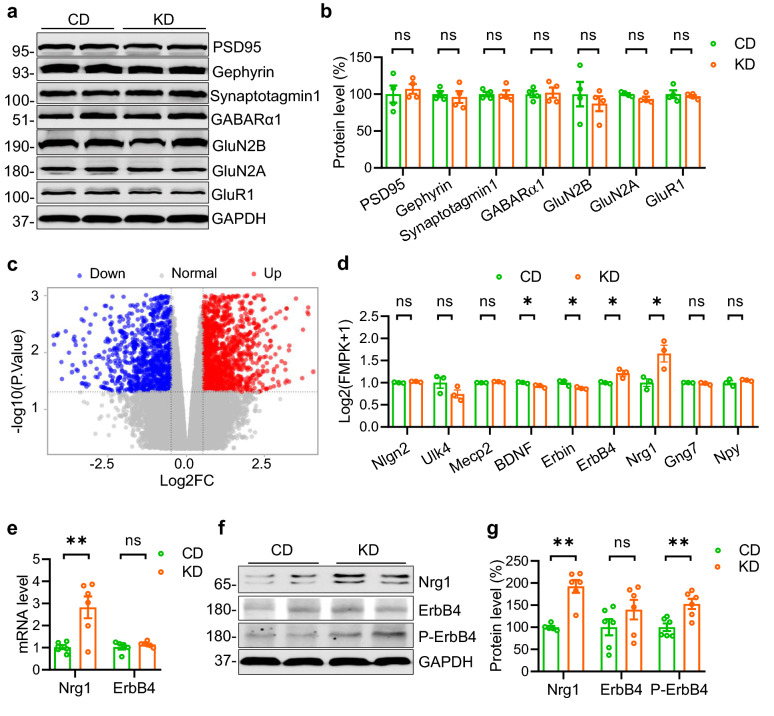
KD treatment elevates Nrg1 expression in the hippocampus. **a** Western blots detecting synaptic proteins in the hippocampus of mice fed with CD or KD, GAPDH serves as the loading control. Shown are representative blots. **b** Quantitative analysis of data in (**a**). n = 4 mice per group. Student’s t test, for PSD95, t_(6)_ = 0.5382, P = 0.6098; for Gephrin, t_(6)_ = 0.4457, P = 0.6714; for Synaptotagmin-1, t_(6)_ = 0.03585, P = 0.9726; for GABARa1, t_(6)_ = 0.2533, P = 0.8085; for GluN2B, t_(6)_ = 0.655, P = 0.5367; for GluN2A, t_(6)_ = 0.1.909, P = 0.1048; for GluR1, t_(6)_ = 0.4986, P = 0.6358. **c** A volcano plot of genes altered by KD treatment in the hippocampus. Genes up-regulated with more than 1.5 fold change with a p-value < 0.05 are depicted in red dots, and those down-regulated with identical fold change and p-value are in green dots. **d** Quantification of several genes that were reported critical for GABAergic signaling from RNAseq data. n = 3 mice per group. Student’s t test, for Nlgn2 (Neuroligin 2), t_(4)_ = 2.259, P = 0.0868; for Ulk4 2, t_(4)_ = 1.653, P = 0.1737; for Mecp2, t_(4)_ = 2.99, P = 0.083; for BDNF, t_(4)_ = 3.378, P = 0.0278; for Erbin, t_(4)_ = 2.917, P = 0.0434; for ErbB4, t_(4)_ = 3.421, P = 0.0267; for Nrg1, t_(4)_ = 3.221, P = 0.0322; for Gng7, t_(4)_ = 1.324, P = 0.256; for Npy, t_(4)_ = 1.162, P = 0.31. **e** The messenger RNA expression level of Nrg1 but not ErbB4 was dramatically increased in the hippocampus. Student’s t test, for Nrg1, n = 6 mice per group, t_(10)_ = 3.608, P = 0.0048; for ErbB4, n = 5 mice for CD group, n = 6 mice for KD group, t_(9)_ = 0.9955, P = 0.3455. **f** Increased expression of Nrg1 and phosphorylated ErbB4 (P-ErbB4) proteins in the hippocampus of mice fed with K.D. Shown were representative blots of two mice from each group. GAPDH served as a loading control. **g** Quantitative analysis of the Western blot data in (**f**). Student’s t test, for NRG1, n = 5 CD-fed mice, n = 6 KD-fed mice, t_(9)_ = 5.735, P = 0.0003; for ErbB4, n = 6 mice per group, t_(10)_ = 1.384, P = 0.1965; for P-ErbB4, t_(10)_ = 3.668, P = 0.0043

The findings that KD preferentially increases inhibitory rather than excitatory synaptic transmission in the hippocampus compelled us to investigate the underlying molecular mechanisms. We found that the levels of synaptic proteins of both excitatory and inhibitory synapses in the hippocampus were comparable between CD and KD groups (Fig. 5a and b), suggesting that the KD may have little effect on the synaptic assemblies. We then carried out transcriptome analysis in the hippocampus of CD-fed and KD-fed mice by whole-genome RNA sequencing. Volcano-plots illustrate differentially expressed genes (DEGs) between CD-fed and KD-fed groups (Fig. 5c). Specifically, there are in total1341 up-regulated genes and 1049 down-regulated genes in the KD-fed mice. We compared a set of genes that were previously reported to regulate GABAergic signaling and found that genes, including *Neuroligin 2*, *Ulk4*, *Mecp2*, *Gng7* and *Npy*, were similar between the two groups (Fig. 5d). Besides, the levels of *BDNF* and *Erbin* genes, which were indicated to promote GABAergic synaptic function, were slightly decreased in KD-fed mice. Notably, the levels of both Nrg1 and ErbB4 were drastically increased in the hippocampus of KD-fed mice (Fig. 5e). Previous studies report that ErbB4 is exclusively expressed in the GABAeric interneuron in the hippocampus [13, 14]. As a ligand, Nrg1 activates ErbB4 to promote GABAergic synaptic transmission [15]. To further validate the transcriptome data, we performed RT-PCR and Western Blot to detect mRNA and protein levels of Nrg1 and ErbB4. As shown in Fig. 5e to g, while *ErbB4* expression was not significantly altered, both the mRNA and protein levels of Nrg1 were dramatically increased in KD-fed mice. Accordingly, the level of phosphorylated ErbB4 was increased (Fig. 5g). These observations demonstrate that KD treatment specifically elevates Nrg1 expression in the hippocampus.

### Knockdown of *ErbB4* from hippocampus diminishes the anti-seizure effects of KD

**Fig. 6:**
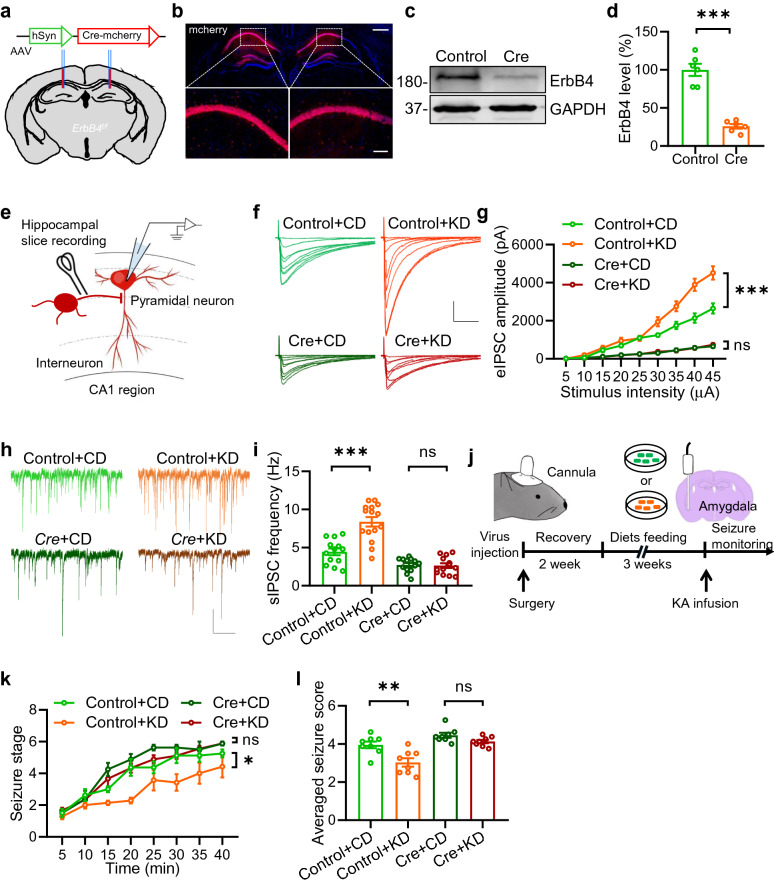
Knockdown of *ErbB4* from hippocampus diminishes the anti-seizure effects of KD. **a** Schematic of virus injection. hSyn promoter-driven Cre virus was injected bilaterally into hippocampal region. **b** Representative images of mcherry expression in virus-injected mice. Hippocampal sections were collected 3 weeks after stereotaxic microinjection of AAV-hSyn-cre-mcherry virus. Enlarged images of the dotted area were shown on the bottom. Scale bar: 500 µm (top) and 100 µm (bottom). **c** Reduced ErbB4 expression in hippocampus of *ErbB4*^*f/f*^ mice injected with AAV-hSyn-Cre-mcherry virus. Shown were representative blots. ErbB4 band density was normalized with the loading control GAPDH. **d** Quantitative analysis of the Western blot data in (**c**). n = 6 mice per group. Student’s t test, t_(10)_ = 8.679, P < 0.0001. **e** Diagram of hippocampal slice recording. Pyramidal neurons in the CA1 region were clamped in whole-cell configuration. Evoked postsynaptic currents were recorded under stimulation by using a concentric bipolar electrode. **f** Representative eIPSC traces. Scale bars, 100 ms and 1000 pA. **g** Knockdown of *ErbB4* in the hippocampus attenuates the effect of KD on eIPSC amplitudes. n = 13 neurons from 3 mice of “Control + CD” group; n = 15 neurons from 3 mice of “Control + KD” group; n = 13 neurons from 3 mice of “Cre + CD” group; n = 13 neurons from 3 mice of “Cre + KD” group. Two-way ANOVA, “Control + CD” vs “Control + KD”: F_(1,234)_ = 51.25, P < 0.0001; “Cre + CD” vs “Cre + KD”: F_(1,216)_ = 0.1648, P = 0.6852. **h** Representative sIPSC traces. Scale bars, 2 s and 50 pA. **i** Knockdown of *ErbB4* in the hippocampus reduces the effect of KD on sIPSC frequency. n = 13 neurons from 3 mice of “Control + CD” group; n = 15 neurons from 3 mice of “Control + KD” group; n = 13 neurons from 3 mice of “Cre + CD” group; n = 13 neurons from 3 mice of “Cre + KD” group. Student’s t test, “Control + CD” vs “Control + KD”: t_(26)_ = 4.979, P < 0.0001; “Cre + CD vs “Cre + KD”: F_(24)_ = 0.2191, P = 0.8284. **j** Diagram of experimental design. AAV virus was injected into hippocampus 2 weeks prior to KD feeding. Three weeks later, mice were subjected to electrophysiological recordings or KA infusion to induce seizures. **k** Knockdown of *ErbB4* in hippocampus diminishes the effect of KD on the development of seizure. Mice were subject to KA infusion and scored for seizure stage every 5 min. n = 8 mice of “Control + CD” group; n = 9 mice of “Control + KD” group; n = 8 mice of “Cre + CD” group; n = 9 mice of “Cre + KD” group. Repeated two-way ANOVA, “Control + CD vs “Control + KD”: F_(1,105)_ = 6.351, P = 0.0235; “Cre + CD vs “Cre + KD”: F_(1,105)_ = 2.382, P = 0.1435. **l** The averaged seizure score is decreased by Knockdown of *ErbB4* in the hippocampus. n = 8 mice of “Control + CD” group; n = 9 mice of “Control + KD” group; n = 8 mice of “Cre + CD” group; n = 9 mice of “Cre + KD” group. Student’s t test, “Control + CD” vs “Control + KD”: t_(15)_ = 3.873, P = 0.0015; “Control + CD” vs “Cre + CD”: t_(14)_ = 2.522, P = 0.0244; “Cre + CD” vs “Cre + KD”: t_(15)_ = 1.733, P = 0.1035; “Control + KD” vs “Cre + KD”: t_(16)_ = 4.771, P = 0.0002

To examine the potential involvement of elevated hippocampal Nrg1 level in the anti-seizure effects of KD, we attempted to knockdown *ErbB4* to block Nrg1/ErbB4 signaling. We first bilaterally injected adeno-associated virus (AAV-hSyn-Cre-mcherry), expressing Cre recombinase and red fluorescent protein-mcherry, into the hippocampus of *floxed-ErbB4* (*ErbB4*^*f/f*^) mice (Fig. 6a) to delete *ErbB4* specifically from hippocampus. The expression of mcherry was confirmed in the hippocampus as shown in Fig. 5b. ErbB4 levels were dramatically reduced in the hippocampus of Cre virus-injected mice compared with controls (Fig. 5c and d). These observations indicate accurate and reliable knockdown of *ErbB4* in the hippocampus. Two weeks after virus injection, mice were subjected to KD feeding for 3 weeks. We then recorded eIPSCs and sIPSCs of pyramidal neurons in the CA1 region (Fig. 5e). Both amplitude of eIPSC and frequency of sIPSCs were increased in KD-fed mice compared with those in CD-fed mice (Fig. 5f–i), which is consistent with previous results. However, there were no differences between Cre-virus-injected mice fed with KD and those fed with CD (Fig. 5f–i), suggesting a critical role of ErbB4 in mediating the effects of KD on inhibitory synaptic transmission. Remarkably, knockdown of *ErbB4* diminished the effect of KD on the development of seizure and the averaged seizure score (Fig. 5j–l). Altogether, these findings demonstrate that Knockdown of *ErbB4* in the hippocampus blocked KD-induced protective effects on synaptic transmission and seizure.

### *ErbB4* in PV^+^ interneurons is necessary for the anti-seizure effects of KD

**Fig. 7:**
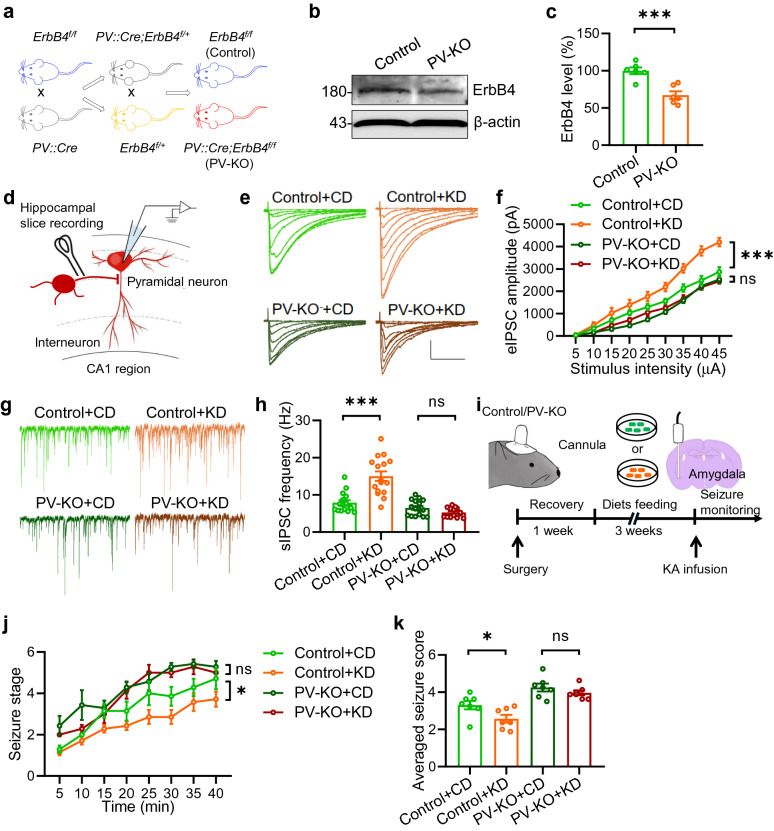
*ErbB4* deletion in PV^+^ interneurons is necessary for the anti-seizure effect of the KD. **a** Mice breeding paradigm. *ErbB4*^*f/f*^ mice were crossed with *PV::Cre* mice; the resulting *PV::Cre;ErbB4*^*f/*+^ mice were crossed with *ErbB4*^*f/*+^ mice to generate PV::Cre;ErbB4 (PV-KO) and *ErbB4*^*f/f*^ (control) mice. **b** Reduced ErbB4 expression in the hippocampus of PV-KO mice. Hippocampal lysates of 2-month-old mice were probed for ErbB4, and GAPDH was taken as a loading control. Shown were representative blots of each genotype. **c** Quantitative analysis of data in (**b**). n = 6 mice per group. Student’s t test, t_(10)_ = 4.853, P = 0.0007. **d** Diagram of hippocampal slice recording. Pyramidal neurons in the CA1 region were clamped in whole-cell configuration. Evoked postsynaptic currents were recorded under stimulation by using a concentric bipolar electrode. **e** Representative eIPSC traces. Scale bars, 100 ms and 1000 pA. **f** Deletion of *ErbB4* in PV^+^ interneurons diminished the effect of KD on eIPSC amplitudes. n = 16 neurons from 3 mice of “Control + CD” group; n = 16 neurons from 3 mice of “Control + KD” group; n = 16 neurons from 3 mice of “PV-KO + CD” group; n = 14 neurons from 3 mice of “PV-KO + KD” group. Two-way ANOVA, “Control + CD” vs “Control + KD”: F_(1,269)_ = 57.97, P < 0.0001; “PV-KO + CD” vs “PV-KO + KD”: F_(1,252)_ = 3.158, P = 0.0768. **g** Representative sIPSC traces. Scale bars, 2 s and 50 pA. **h** Deletion of *ErbB4* in PV^+^ interneurons diminished the effect of KD on sIPSC frequency. n = 16 neurons from 3 mice of “Control + CD” group; n = 16 neurons from 3 mice of “Control + KD” group; n = 16 neurons from 3 mice of “PV-KO + CD” group; n = 14 neurons from 3 mice of “PV-KO + KD” group. Student’s t test, “Control + CD” vs “Control + KD”: t_(30)_ = 4.985, P < 0.0001; “PV-KO + KD” vs “PV-KO + KD”: t_(28)_ = 1.873, P = 0.0715. **i** Schematic of experimental design. Control and PV-KO mice that were feed with CD or KD for 3 weeks were subject to KA infusion to induce seizures. **j** The development of seizure with time following KA infusion. n = 7 mice for each group. Repeated two-way ANOVA, “Control + CD” vs “Control + KD”: F_(1,12)_ = 5.685, P = 0.0345; “PV-KO + CD” vs “PV-KO + KD”: F_(1,12)_ = 1.311, P = 0.2746. **k** *ErbB4* deletion in PV^+^ interneurons diminished the effect of KD on the averaged seizure score. n = 7 mice for each group. Student’s t test, “Control + CD” vs “Control + KD”: t_(12)_ = 2.384, P = 0.0345; “PV-KO + CD” vs “PV-KO + KD”: t_(12)_ = 1.145, P = 0.2746

Previous studies report that ErbB4 mainly expresses in PV^+^ interneurons in the brain [13, 16, 17]. We crossed *PV-Cre* mice, where the expression of Cre is driven by *PV* gene promoter [17], with floxed *ErbB4* (Control) mice to obtain *PV-ErbB4*^*–/–*^ mice (abbreviated to PV-KO, hereafter) (Fig. 7a). ErbB4 levels were dramatically attenuated in PV-KO mice compared with those in control mice (Fig. 7b and c). We recorded eIPSCs and sIPSCs of pyramidal neurons in the CA1 region of mice fed with the CD or KD (Fig. 7d). Both amplitudes of eIPSC and frequency of sIPSCs were increased in KD-fed control mice when compared with those in CD-fed control mice, which is consistent with previous results. However, *ErbB4* knockout in the PV^+^ interneuron dramatically diminished their difference (Fig. 7e to h). These data suggest an indispensable role of ErbB4 in PV^+^ interneurons in mediating the effects of KD on inhibitory synaptic transmission. Remarkably, In the seizure induction experiments (Fig. 7i)), deletion of *ErbB4* in PV^+^ neurons diminished the impact of KD on the development of seizures as well as the averaged seizure scores (Fig. 7j and k). Altogether, these findings corroborate the crucial role of ErbB4 in PV^+^ interneurons in mediating the effects of the KD on synaptic transmission and seizure.

## Discussion

Our study identifies a novel role of Nrg1/ErbB4 signaling in mediating the anti-seizure effects of the KD and offers an explanation to increased GABA level in epileptic animal models and human patients with KD treatment. These findings provide insight into not only pathophysiological functions of Nrg1/ErbB4 signaling in epilepsy but also indicate that such signaling mechanism can be therapeutically manipulated to treat refractory epilepsy.

KD therapy has been approved to be an effective treatment for epilepsy. Yet, the exact underlying mechanisms remain obscure. Numerous studies have reported that the GABA level is increased explicitly in the brains of humans and animal models with KD treatment [8–10]. In light of the critical role of GABAergic signaling in controlling seizures, understanding the molecular regulatory mechanisms will facilitate the development of precise interventions that lack adverse effects of the KD. In the hippocampus, GABAergic transmission is tightly regulated by Nrg1/ErbB4 signaling [15]. EbrB4 is exclusively expressed in GABAergic interneurons in the cortex and hippocampus [14]. Activation of ErbB4 by its ligand-Nrg1 increased the probability of GABA release from the axonal terminal [13, 17]. Interestingly, alteration of Nrg1/ErbB4 signaling is implicated in epilepsy animal models and human patients [18–20], and disruption of Nrg1/ErbB4 signaling pharmacologically and/or genetically promotes kindling progression, which results in increased spontaneous seizures in kindling epilepsy model [18]. These reports reveal a critical role of Nrg1/ErbB4 signaling in the regulation of epileptogenesis.

Our results provide compelling evidence supporting the hypothesis that Nrg1/ErbB4 signaling is pivotal for the anti-seizure effects of the KD. By using multiple techniques including RNAseq, RT-PCR and Western blot, we demonstrate that Nrg1 expression is dramatically elevated in the hippocampus of mice fed with the KD; Moreover, *ErbB4* deletion in the hippocampus or PV^+^ interneurons abolishes the effects of the KD on both GABAergic activity and seizures. Collectively, these findings indicate that Nrg1/ErbB4 signaling in the hippocampus is indispensable for the anti-seizure effect of KD. However, it is not clear how Nrg1/ErbB4 signaling regulates GABA release.

Previous reports have proposed several potential downstream targets for Nrg1/ErbB4 signaling, including voltage-gated potassium channel Kv1.1, which mediate the effect of Nrg1/ErbB4 signaling on the firing of PV^+^ interneurons in the cortex [19]; voltage-gated sodium channel, whose activity in ErbB4^+^ neurons was reduced by Nrg1 treatment in vitro [21]; transient outward channel Kv4.2, whose expression was increased by Nrg1/ErbB4 signaling through the Akt/mTOR pathway in cultured rat cerebellar granule neurons [22]. Future studies are needed to verify whether such mechanisms underlie the role of Nrg1/ErbB4 signaling in the anti-seizure effects of KD.

Recent studies have identified several electrical modulators affected by the KD, including ATP-sensitive K^+^ channels [23], voltage-dependent Ca^2+^ channels [24], AMPA-type glutamate receptors [25], adenosine A1 receptors [26], vesicular glutamate transporters [27], lactate dehydrogenase [28]. In these reports, KD treatment downregulated either the excitability of pyramidal neurons or the excitatory synaptic transmission. However, some other studies also indicated an unaltered glutamate level in both rodent animal models and human patients with KD treatment [9, 10]. While the reason for the discrepancy is not clear, we examined the excitatory synaptic transmission on the SC-CA1 synapses and found that neither eEPSC nor sEPSCs was changed between mice fed with the KD and those fed with the CD, implicating minimal effects of the KD on excitatory synaptic transmission, specifically (if not all) on SC-CA1 synapses. It is noteworthy that, Nrg1/ErbB4 signaling in the hippocampus and amygdala at the adult stage exhibits little effect on excitatory synaptic transmission [13, 17], which is in accord with our observations. Based on these results, we postulate that the KD alters inhibition/excitation balance by increasing GABA release through up-regulation of Nrg1/ErbB4 signaling, which eventually influences seizure threshold and epileptogenesis. Nevertheless, we cannot exclude the possibility that those proposed molecular targets described above may also involve in the regulation of Nrg1/ErbB4 signaling. Future studies are warranted to investigate the potential interactions among these factors.

It is not clear so far about the underlying mechanisms through which KD treatment increases Nrg1 expression. Previous studies have indicated that dysregulated epigenetic changes, including acetylation of histones, play a prominent role in regulating epileptogenesis [29]. Notably, a well-known anti-epileptic drug-valproic acid (VPA), also an inhibitor of histone deacetylases (HDACs), increases H3 acetylation in the brain [30], eventually suppresses seizures by increasing the levels of GABA in the brain. These findings support the disease-modifying effect of histone acetylation status. More importantly, β-hydroxybutyrate, the main form of ketone bodies, is reported to increase histone acetylation and serves as an endogenous HDAC inhibitor [31]. Indeed, several studies have reported the beneficial effects of KD/ketone bodies on oxidative stress and spatial memory, probably through acetylation regulation [32, 33]. It is interesting to investigate in the future whether KD affects Nrg1 expression via histone acetylation and the underlying regulatory mechanisms. Nevertheless, our study demonstrates an important role of Nrg1/ErbB4 signaling in mediating the anti-seizure effects of KD and provide insight into the epilepsy treatment.

## Materials and methods

### Reagents and antibodies

Chemicals were purchased from Sigma-Aldrich unless otherwise indicated. DL-AP5 (0105), CNQX (0190) were purchased from Tocris Bioscience. Following antibodies were used: Mouse anti-Nrg1 (Santa Cruz Biotechnology) (sc-393006; 1:1000 for blotting); Mouse anti-ErbB4 (Santa Cruz Biotechnology) (sc-8050; 1:1000 for blotting); Rabbit anti-P-ErbB4 (Cell Signaling Technology) (Tyr1284; 1:1000 for blotting); Mouse anti-GAPDH (Santa Cruz Biotechnology) (sc-32233; 1:1000 for blotting); Rabbit anti-PSD95 (Cell Signaling Technology) (#3450; 1:1000 for blotting); Mouse anti-Gephyrin (Santa Cruz Biotechnology) (sc-25311; 1:1000 for blotting); Rabbit anti-Synaptotagmin-1 (Cell Signaling Technology) (#14,558; 1:1000 for blotting); Rabbit anti-GABARa1 (Millipore) (3,108,661; 1:1000 for blotting); Rabbit anti-GluN2B(NMDAR2B) (Cell Signaling Technology) (#14,544; 1:1000 for blotting); Rabbit anti-GluN2A (Cell Signaling Technology) (#4205; 1:1000 for blotting); Rabbit anti- GluR1 (Cell Signaling Technology) (#13,185; 1:1000 for blotting).

### Animals

Eight-to twelve-week-old male mice were used for experiments. The detailed information about mouse strains has been described previously: *ErbB4*^*f/f*^ [17]; *PV::Cre* [16]. Genotyping primer sequences were as follows: *ErbB4*^*f/f*^, 5′-AAA TCA TCC TCT TGT GTG CTT TTG TAC-3′ and 5′-CTC GGT ACT GCT GTT CCA GGT CAG A-3′; *PV::Cre*, 5′-AAA TGC TTC TGT CCG TTT GC-3′ and 5′-CAG AGC AGG CAT GGT GAC TA-3′ and 5′-AGT ACC AAG CAG GCA GGA GA-3′ and 5′-ATG TTT AGC TGG CCC AAA TG-3′. In all experiments, significant efforts are made to minimize the total number of animals used while maintaining statistically valid group numbers. Animal housing conditions were maintained at a temperature of 22 ± 1 °C, at > 30% humidity and a standard 12 h light/dark cycle. All animal experimental protocols were approved by the Animal Ethics Committee of Guangzhou Medical University.

### Diets and feeding

The contents of control diet (CD) and ketogenic diet (KD) (Research Diets) are described previously [34]. Briefly, CD (D10070802) (per-calorie macronutrient) contained: 10% protein, 80% carbohydrates, and 10% fat; KD (D10070801) was constituted by: 10% protein and 90% fat. Sources of fat are Soybean oil and cocoa butter. Micronutrient content, fiber, and preservatives are matched according to a per-calorie basis. During experiments, CD or KD was placed in the food well of the cage-top wire lid (stick-like texture). Diets were changed every week.

### Blood ketones and glucose

Blood ketone levels were measured using the blood glucose and ketone monitoring system (FreeStyle Optium Neo, Abbott) according to the manufacturer’s instructions. Briefly, after sterilization with 70% ethanol, the tail tips of mice under tests were cut using a clean scissor, and a drop of blood was collected. Using the test strip (Abbott), levels of β-hydroxybutyrate or glucose were determined.

### Cannula implantation

Adult male mice were maintained anesthetized with isoflurane (2–3%) and head-fixed in a stereotaxic device (RWD Life Science.Inc). After an incision was made in the scalp, a small hole was drilled into the skull, and a guide cannula (IO: 0.48 mm; RWD Life Science.Inc) was implanted inside the right amygdala (coordinates: anteroposterior, –1.22 mm; dorsoventral, –4.5 mm; mediolateral, 3 mm relative to bregma) and cemented onto the skull with dental cement. Mice were then returned to their homecages for at least 1 week.

### Seizure induction and behavioral monitoring

For kainic acid (KA)-induced acute seizure model, mice with guide cannulas were gently restrained and an infusion cannula (IO: 0.3 mm; RWD Life Science.Inc) was inserted into the amygdala through the guide cannula. 0.15 µl of KA (3 mg/ml) was infused into the amygdala at the flow rate of 2 nl/s controlled by microinjector (NanojectIII, Drummond Scientific). The cannula was kept in the right amygdala for two additional min after completion of infusion and withdrew slowly to minimize reflux along the injection tract. Behavioral seizures were classified based on the criteria described by Racine [35] and scored every 5 min by a blinded investigator: stage 0, no seizure; stage 1, arrest and rigid posture; stage 2, head nodding; stage 3, sporadic full-body shaking, spasms; stage 4, chronic full-body spasms; stage 5, jumping, shrieking, falling over; stage 6, violent convulsions or death.

For KA-induced spontaneous recurrent seizure (SRS) model, diazepam (8 mg kg^−1^) was i.p. injected 1 h after status epilepticus (SE) induction. The food for mice was switched from standard chow diet to CD and KD, respectively. Three weeks later, mice were video-monitored from 8 am to 8 pm each day for 2 weeks. SRS was defined with score ≥ 4 and counted by review of video files by a blinded investigator.

### Timm staining

We performed Timm staining using FD Rapid TimmStain™ Kit (Biosensis) and followed manufacturer’s instruction. Images were randomly taken from dorsal hippocampus with microscope (CTR6, Leica). Staining intensities were quantified using image J. The extent of mossy fiber sprouting was quantified by Timm index, which denote the ratio between total area of Timm granules and total dentate gyrus length [18].

### Electrophysiological recording

Adult male mice were anesthetized with isoflurane. Brains were quickly removed and chilled in ice-cold modified artificial cerebrospinal fluid (ACSF) containing (in mM): 120 Choline-Cl, 2.5 KCl, 7 MgCl_2_, 0.5 CaCl_2_, 1.25 NaH_2_PO_4_, 25 NaHCO_3_, and 10 glucose. Coronal hippocampal slices (300 µm) were sectioned in ice-cold modified ACSF using a VT-1000S vibratome (Leica, Germany) and transferred to a storage chamber containing regular ACSF (in mM) (126 NaCl, 3 KCl, 1 MgSO_4_, 2 CaCl_2_, 1.25 NaH_2_PO_4_, 26 NaHCO_3_, and 10 glucose) at 32 °C for 30 min and at room temperature (24 ± 1 °C) for additional 1 h before recording. All solutions were saturated with 95%O_2_/5%CO_2_ (vol/vol).

Slices were placed in the recording chamber superfused (2 ml/min) with ACSF. Whole-cell patch-clamp recording from CA1 pyramidal neurons was visualized with infrared optics using an upright microscope equipped with an infrared-sensitive CCD camera (DAGE-MTI, IR-1000E). Pipettes were pulled by a micropipette puller (P-97, Sutter instrument) with a resistance of 3–5 MΩ. Recordings were made with MultiClamp 700B amplifier and 1440A digitizer (Molecular Device).

For spontaneous excitatory postsynaptic current (sEPSC) recording, pyramidal neurons were held at −70 mV in the presence of 20 μM RS-95531, with the pipette solution containing (in mM): 125 Cs-methanesulfonate, 5 CsCl, 10 Hepes, 0.2 EGTA, 1 MgCl_2_, 4 Mg-ATP, 0.3 Na-GTP, 10 phosphocreatine and 5 QX314 (pH 7.40, 285 mOsm). Evoked excitatory postsynaptic currents (eEPSCs) were recorded by stimulating Schaffer Collaterals (SC)-CA1 pathway with gradually increasing stimulation intensities by using a concentric bipolar electrode (FHC), ~ 200 µm away from recording pipette.

To record spontaneous inhibitory postsynaptic current (sIPSC), pyramidal neurons were held at −70 mV in the presence of 20 μM CNQX and 100 μM AP-5, with the pipette solution containing (in mM): 140 CsCl, 10 Hepes, 0.2 EGTA, 1 MgCl_2_, 4 Mg-ATP, 0.3 Na-GTP, 10 phosphocreatine and 5 QX314 (pH 7.40, 285 mOsm). To measure evoked inhibitory postsynaptic currents (eIPSCs), stimulation electrode was positioned on the Schaffer Collaterals (SC)-CA1 pathway, ~ 100 µm away from recording pipette.

In all experiments, series resistance was maintained below 20 MΩ and not compensated. Cells would be rejected if membrane potentials were positive more than −60 mV; or if series resistance fluctuated more than 20% of initial values. Data were filtered at 1 kHz and sampled at 10 kHz.

### Western blot

Brain tissue homogenates were prepared in RIPA Buffer containing (in mM): 50 Tris–HCl, pH 7.4, 150 NaCl, 2 EDTA, 1 PMSF, 50 sodium fluoride, 1 sodium vanadate, 1 DTT with 1% sodium deoxycholate, 1% SDS and 1% protease inhibitors cocktails. Samples were resolved on SDS/PAGE and transferred to nitrocellulose membranes, which were incubated in TBS buffer containing 0.1% Tween-20 and 5% milk for 1 h at room temperature before incubating with primary antibodies (overnight at 4 °C). After wash, the membranes were incubated with HRP-conjugated secondary antibodies (Absin ImmunoResearch) in the same TBS buffer for 1 h at room temperature. Immunoreactive complex bands were visualized using enhanced chemiluminescence (Pierce) and captured using the Genesys imaging system (Gene Company Limited, UK). Band densities of interested proteins were normalized with loading control.

### RNA sequencing and transcriptome analysis

Total RNA was extracted from hippocampi using Trizol (Life Technologies, Carlsbad, CA, USA). mRNA was purified from total RNA using poly-T oligo-attached magnetic beads. Fragmentation was carried out using divalent cations under elevated temperature in NEBNext First Strand Synthesis Reaction Buffer(5X). First-strand cDNA was synthesized using random hexamer primer and M-MuLV Reverse Transcriptase (RNase H-). Second strand cDNA synthesis was subsequently performed using DNA Polymerase I and RNase H. Remaining overhangs were converted into blunt ends via exonuclease/polymerase activities. After adenylation of 3′ ends of DNA fragments, NEBNext Adaptor with hairpin loop structure was ligated to prepare for hybridization. To select cDNA fragments of preferentially 250–300 bp in length, the library fragments were purified with AMPure XP system (Beckman Coulter, Beverly, USA). Then 3 µl USER Enzyme (NEB, USA) was used with size-selected, adaptor-ligated cDNA at 37 °C for 15 min followed by 5 min at 95 °C before PCR. Then PCR was performed with Phusion High-Fidelity DNA polymerase, Universal PCR primers and Index (X) Primer. At last, PCR products were purified (AMPure XP system) and library quality was assessed on the Agilent Bioanalyzer 2100 system.

The library preparations were sequenced on an Illumina Hiseq 4000 and 150 bp paired-end reads were generated. Image analysis and base calling were performed with Illumina CASAVA pipeline, and finally 150 bp paired-end reads were generated. Feature Counts v1.5.0-p3 was used to count the reads numbers mapped to each gene. And then FPKM of each gene was calculated based on the length of the gene and reads count mapped to this gene. Differential expression analysis was performed using the DESeq2 R package (1.10.1). Genes with an P-value < 0.05 found by DESeq2 were assigned as differentially expressed. Note that the raw sequencing data have been deposited in the Gene Expression Omnibus (GEO) database (GSE156239).

### qRT-PCR analysis

qRT-PCR was performed as described previously [34]. Briefly, total RNA was isolated by using TRIzol reagent (15596–026, Invitrogen). RNA (1 µg) was reverse-transcribed with oligo dT-primers using Maxima reverse transcriptase (EP 0742, Fermentas) followed by q-PCR with SYBR Green detection (K 0222, Fermentas). Samples were assayed in triplicates, with each plate having loading standards in duplicate. RNA levels of *ErbB4* and *Nrg1* were normalized to those of GAPDH. Primer sequences were: ErbB4, 5′-CAT GGC CTT CCA ACA TGA CTC TGG-3′ and 5′-GGC AGT GAT TTT CTG TGG GTC CC-3′; Nrg1, 5′-ATG TGC AAA GTG ATC AGC AAG -3′ and 5′-TGA GGA CAC ATA GGG TCT TT -3′; GAPDH, 5′-GGT TGT CTC CTG CGA CTT CA-3′ and 5′-CCA CCA CCC TGT TGC TGT AG -3′.

### Immunofluorescent staining

Anesthetized mice were transcardially perfused with PBS followed by 4% PFA. Removed brains were fixed in 4% PFA at 4 °C for 8 h. After dehydration by 30% sucrose, brain blocks were frozen and cut into 30-μm-thick sections on a cryostat (CM1950, Leica). Sections were permeabilized with 0.3% Triton X-100 and 5% BSA in PBS and incubated with primary antibodies at 4 °C overnight. After washing with PBS for 3 times, samples were incubated with Alexa Fluor-conjugated secondary antibodies (1:800, Jackson ImmunoResearch) for 1 h at room temperature. Samples were mounted with Vectashield mounting medium (Vector lab) and images were captured using Leica immunofluorescent microscope (CTR6, Leica).

### Experimental design and statistical analysis

Adult male mice (≥ 2 months) were used in the present study. Animal or replicate numbers for each experiment and results of the statistical analyses, including degrees of freedom and exact p-values were mentioned in the figure legends. Statistical analyses were performed using GraphPad Prism version 8.0.1 (GraphPad Software). Sample size choice was made based on previous studies [36, 37]. Student’s t test was used to compare data from two groups. Repeated two-way ANOVA was for KA-induced seizure development studies. Regular two-way ANOVA was used for electrophysiological studies that analyze more than two parameters. All tests were two-sided. All data represent mean ± SEM, unless otherwise stated. P < 0.05 was considered to be statistically significant.
